# Processing Code-Switching in Algerian Bilinguals: Effects of Language Use and Semantic Expectancy

**DOI:** 10.3389/fpsyg.2016.00248

**Published:** 2016-03-01

**Authors:** Souad Kheder, Edith Kaan

**Affiliations:** Department of Linguistics, University of Florida, GainesvilleFL, USA

**Keywords:** code-switching, semantic constraint, base language, language expectancy, processing, listening

## Abstract

Using a cross-modal naming paradigm this study investigated the effect of sentence constraint and language use on the expectancy of a language switch during listening comprehension. Sixty-five Algerian bilinguals who habitually code-switch between Algerian Arabic and French (AA-FR) but not between Standard Arabic and French (SA-FR) listened to sentence fragments and named a visually presented French target NP out loud. Participants’ speech onset times were recorded. The sentence context was either highly semantically constraining toward the French NP or not. The language of the sentence context was either in Algerian Arabic or in Standard Arabic, but the target NP was always in French, thus creating two code-switching contexts: a typical and recurrent code-switching context (AA-FR) and a non-typical code-switching context (SA-FR). Results revealed a semantic constraint effect indicating that the French switches were easier to process in the high compared to the low-constraint context. In addition, the effect size of semantic constraint was significant in the more typical code-switching context (AA-FR) suggesting that language use influences the processing of switching between languages. The effect of semantic constraint was also modulated by code-switching habits and the proficiency of L2 French. Semantic constraint was reduced in bilinguals who frequently code-switch and in bilinguals with high proficiency in French. Results are discussed with regards to the bilingual interactive activation model (Dijkstra and Van Heuven, 2002) and the control process model of code-switching (Green and Wei, 2014).

## Introduction

Some bilingual speakers may daily interact in a context where similar speakers use both languages in the same conversation or even in the same utterance of speech. This type of interaction is usually known as code-switching (CS). During code-switching, bilinguals may be listening to an utterance that starts in their first language (L1) but may or may not end in that same language depending on the speaker’s speech planning (e.g., Kroll and Gollan, 2014). How does a bilingual listener integrate an item from their second language (L2) while listening to L1? While the choice of code-switching is made by the speaker, this choice may still impact the listener. Listeners are active recipients who constantly make inferences about the speakers’ intentions, and may develop models on how they may respond (Gross, 2000; Green and Abutalebi, 2013). We know from studies which tested bilingual speakers during reading in L1 or in L2 that the semantic and syntactic context of a sentence speeds up word recognition, assumingly through reducing the number of the activated candidates. Words embedded in a semantic context that is highly constraining are processed faster than words embedded in a semantic context that is neutral (e.g., Schwartz and Kroll, 2006; Duyck et al., 2007; van Hell and de Groot, 2008; Van Assche et al., 2009, 2011; Titone et al., 2011). Studies which explored sentential context influence during switching mainly concerned reading from L2 to L1. While code-switching is primarily conversational where bilinguals may either be speakers (production) or listeners (comprehension), relatively fewer studies focused on listening comprehension. Furthermore, previous studies often failed to give details concerning the switching behavior of their participants in their daily lives. Hence, the populations tested previously may have included speakers who did not switch frequently in natural conversations. The daily use of the bilingual languages and their different interactional contexts, may have a role in shaping the use of sentence constraints. In multilinguals, code switching may be more frequent in one language combination than another. Thus, it is important to explore sentential effects during code-switching taking into account the daily language use of the bilinguals (e.g., Green, 2011). The main objective of the current study is to determine to what extent (1) the language that precedes a code-switch, and (2) the semantic context affect the expectancy of a language switch during listening to L1, and to see if this effect is modulated by (3) code-switching habits and (4) the L2 proficiency of the bilinguals.

### Effects of Semantic Constraint

Altarriba et al. (1996) explored the effect of semantic context constraints in mixed-language sentences in Spanish–English bilinguals during reading for comprehension. Their goal was to determine whether sentence context effects can extend to the determination of the lexical features. Participants read Spanish and English target words inserted in English low and high constraint sentences while fixations were measured with an eye tracker. The results revealed that high frequency Spanish words produced slower naming times and longer fixation times when they appeared in high constraint English sentences but not in a low constraint context. This suggests that in the highly constrained context the readers generated semantic and lexical features of the upcoming words in the English context. When the target word appeared in Spanish, not the language of the context, the expectations regarding the lexical features were not met. The readers expected a word form with a specific meaning, suggesting that semantic context can selectively activate a word in one language.

The effect of sentence context in code-switched sentences was also found in studies using event-related potentials (ERPs; e.g., Moreno et al., 2002; Proverbio et al., 2004). Moreno et al. (2002) explored the ERPs to a language switch when English–Spanish bilinguals read both moderately constraining and highly constraining English sentences. The sentences appeared in three conditions: either ending with expected English words, ending with their Spanish translations (code-switches), or ending with English synonyms (lexical switches). Code-switches elicited a large posterior positivity in both context types usually associated with the processing of an unexpected or improbable event. The authors suggested that the Spanish words acted like improbable events probably because they occurred in written text and were from English to Spanish while natural code-switching occurs mostly in speaking and is from Spanish to English. Moreno et al. (2002), thus, suggested that more natural code-switches, would reduce the positivity making the code-switch less improbable. In Proverbio et al. (2004), Italian professional interpreters evaluated whether a sentence-final word made sense with the rest of the sentence in a unilingual or a mixed/code-switched condition. Sentences ended in an unexpected final word that created a semantic incongruence or in a highly probable and congruent word. Even when the switch was entirely predictable the mixed conditions revealed processing cost. A larger N400, associated with semantic integration, was reported for the final words in the mixed compared to the unmixed sentences. Although the participants in these studies were highly proficient, the authors did not report to what extent these participants mixed languages in daily conversation.

In a cross-modal naming (CMN) paradigm (i.e., naming a visual word in an auditory context) Hernández et al. (1996) explored the effect of expectancy and predictability in sentential priming within and between languages during listening. Spanish–English bilinguals heard sentences and named a word that appeared on the screen at a certain point during the presentation of the sentence. The target word was either related or unrelated to a critical word in the sentence and was either presented immediately or delayed. In the mixed condition (English–Spanish or Spanish–English sentences), facilitation occurred only under the delayed naming condition. The results showed that cross-language priming, that is semantic and lexical facilitation, appears when participants expect the language of the target word, when they have sufficient time to generate a response, that is, to access and integrate the target word in the sentence context, or both. In addition, the results also showed that priming was larger for English than for Spanish suggesting that priming is more robust for the language that bilinguals are using most often in their everyday lives. Hernández et al. (1996) reported that the participants in this study were native speakers of Spanish who were immersed in an English environment since they started school and thus may have been English-dominant. While it is possible that English dominated in the participants’ speech, they may or may not be from a code-switching community.

Cieślicka and Heredia (2015) used CMN to investigate the effect of context and cross-language priming on lexical access in Spanish–English bilinguals. Participants listened to sentences in Spanish or English that contained a prime (e.g., *war*) and then named a target word either in Spanish or in English. The target was either related (e.g., *peace*) or unrelated (e.g., *boca*) to the prime. In addition, sentence context was either biasing or not toward the target prime. The targets were presented at the offset of the prime to examine the activation of L1 after the processing of L2 and vice versa. When the context language was Spanish, context manipulation did not modulate priming effect between within and cross-language conditions, but it affected the overall processing of the target words in the Spanish–English conditions. The targets in the biased context were faster to name than those in the unbiased context. However, when the context language was English, cross-language priming effect was greater than within-language priming effect and biased context slowed down the naming of the target words. An important finding in this study was the language asymmetry in lexical access. Participants were faster to respond to words in the L2 when the preceding context was in the L1 than vice versa. The interpretation Cieślicka and Heredia (2015) suggested was that with increased proficiency in L2, the ease with which bilinguals access words in their languages depends on language usage.

### Code Switching Habits and Effect of Base-Language

The overall results from the above studies suggest that semantic context as well as the language of the preceding sentence affect the processing of upcoming words in bilingual speakers. However, different bilinguals may have different linguistic experiences that have prepared them as speakers/listeners to use one or the other language separately in one context, or even use both in the same utterance in another context (e.g., Grosjean, 2008). Meuter (2009) sees the ability to select among these options as a sign of highly proficient language use and goal-directed behavior but at the same time dictated by communicative contexts. Specifically, in a code-switching context, bilinguals must be ready to integrate (comprehension) and respond (production) in any language, while being sensitive to the cues of each language such as accent and language-specific morpho-syntactic patterns. Thus, processing a language switch may depend greatly on language use and switching habits. For instance in an eye tracking study, Valdés Kroff et al. (in press) found that the masculine Spanish article *el* functioned as a default article with English nouns in Spanish–English code-switching resulting in competitor effects due to phonological competition over the feminine article *la*. When the Spanish article + English noun code-switches were tested with bilinguals who were not code-switchers (Valdés Kroff et al., 2011), a processing delay was found for both *el* and *la* with English noun combinations. Results from these studies suggest that bilinguals who are exposed to code-switching showed a pattern of processing code-switches that was different from the bilinguals who were not exposed to code-switching. The latter group suffered greater processing costs when they encountered code-switches.

There has been an increased focus on language use and switching habits of the bilinguals and their effect on switching control processing. Green and Wei (2014) have proposed a control process model based on the adaptive control hypothesis (Green and Abutalebi, 2013). The adaptive control hypothesis proposes a language control system where processes vary with different interactional contexts. Three different interactional contexts are identified in a bilingual setting (Green and Abutalebi, 2013; Green and Wei, 2014). In a “single language context” one language is used in one context (e.g., at home) and the other used in another context (e.g., at school/work). In a “dual language context,” both languages may be used in the same environment but with different speakers. Finally, in a “dense code-switching context” the bilinguals habitually switch between their languages within the same utterance and adapt words from one language to fit within the structure of the other. Therefore, in the dual context there is higher demand on processes that control interference in order to minimize inappropriate switching. The language schemas are in a competitive relationship and control alternates between the schemas of the different languages. In the dense code-switching context, there is increased demand on control processes that allow alternative forms. Hence, language task schemas cooperate to allow alternative forms depending on their appropriateness in the given context. The hypothesis states that different contexts of language use may shape the adaptation of control processes, i.e., a bilingual’s experience using both languages will shape the specific cognitive mechanisms that more generally support bilingual language control. The control system adapts to the demands of the different interactions to avoid or reduce the interactional cost that may arise when bilinguals from different interactional contexts converse. For instance, when bilinguals who code-switch feel the necessity to avoid code-switching in a given interaction due to inappropriateness, they face an interactional cost. This is because this interaction imposes on them to remain in one language and block interference from the other. To control for interference they have to engage some control processes in which they are not well-trained and this incurs extra processing load.

When dense code-switching is not a common language practice of the bilinguals, it is likely that encountering a lexical switch within the same utterance imposes higher cognitive processing demands on control processes in which these bilinguals are not well-trained. Similarly, when bilinguals from a dense code-switching context encounter code-switches that do not allow alternative forms (adaptation), they are forced to use competitive control processes not typical of their language processing. We may speculate that the conflicting results reported in previous studies concerning the ease of code-switching may be due to incongruities between the participants’ habitual interactional contexts and the type of code-switched material on which they have been tested.

Bilinguals who code-switch may also differ in respect to the direction of code-switching, that is, the language they switch from or the “base language.” Many code-switching studies examined switching from L2 to L1. Although this switching pattern is attested among bilingual communities, it is less common than switching from L1 to L2 (e.g., Moreno et al., 2002). For instance Spanish–English bilinguals in Texas code-switch more to their L2 English when they communicate in their L1 Spanish than they do to L1 Spanish when they communicate in L2 English (Heredia and Altarriba, 2001). Additionally, in multilingual communities in which speakers may use more than two languages, switching may involve typically one pair of the languages but not the other. Since switching between languages is conversational, it is likely that the languages that are used in everyday conversations are the ones which are involved in code-switching. In the present study we examined the effect of semantic constraint and the language preceding the switch on processing code-switching by comparing two types of mixed-language sentences, one which typically occurs in everyday conversations and the other which does not. We explored Algerian bilinguals who belong to a community where code-switching is a well-established way of communication (e.g., Boumans and Caubet, 2000) but who differ in the frequency of daily code-switching.

### The Current Study

The current experiment examined the effect of context language (base language), semantic constraints and language use on the expectancy of a language switch during listening comprehension. The habit and frequency of switching between a pair of languages rather than another may affect lexical expectancy and switching licensing. Code-switching between Algerian Arabic (AA) and French (FR) is conversational and frequent among some Algerian bilinguals but not code-switching between Standard Arabic (SA) and French. One of the possible reasons for this distribution is that although Algerians are introduced to Standard Arabic from the time they start school, and sometimes earlier, it is considered a school language used to study text books and get knowledge. Standard Arabic is never heard in conversations in the street or even in classrooms between students themselves. It is typical, however, to hear Algerian bilinguals speak Algerian Arabic and include French switches of varying length and morphological adaptation to Algerian Arabic structure (Boumans and Caubet, 2000; Bentahila and Davies, 2001, 2002).

We compared Algerian Arabic-French (AA-FR) code-switching to Standard Arabic-French (SA-FR) code-switching to investigate whether a language as a whole (base language) plays the role of a cue in expecting a language switch. In other words, when switching between a pair of languages that is typically attested in natural code-switching (AA-FR), Algerian bilinguals may expect a switch to French when they hear Algerian Arabic. However, when switching between a pair that is not so typical (SA-FR) Algerian bilinguals may not expect a language switch. In the latter case, code-switches may be harder to process and integrate with the preceding context than in the former case.

The second goal of this study was to examine whether the semantic constraints of the sentence context affects the expectancy of a language switch, and to compare the semantic constraints effect when switching between languages is typical (AA-FR) and when switching between languages is not typical (SA-FR). A high constraint context provides semantic cues that bias toward a specific lexical item and possibly its language (e.g., Altarriba et al., 1996; DeLong et al., 2005). On the other hand, a switch may be unexpected and hard to process even when it is highly predictable (Moreno et al., 2002; Proverbio et al., 2004). Accordingly, if a switch is unexpected in a more probable context (AA-FR) code switching, it should be more unexpected in a less probable context (SA-FR). Results from another experiment on Algerian bilinguals (Kheder et al., in preparation) that examined switch costs in high and low constraint contexts revealed semantic effects, that is, facilitation effect in high compared to low-constraint contexts regardless of word frequency, in both unilingual French context and AA-FR code-switching (as opposed to the Altarriba et al., 1996, in which context effects disappeared in high-constraint context for frequent target words). Based on the above, we predicted a language switch to be more expected in the high-constraint context than in the low-constraint context. In addition, we expected the effect of semantic constraint to be larger when switching from Algerian Arabic base language than when switching from Standard Arabic.

The third goal was to see whether language expectancy in code-switching was affected by the bilingual’s switching habits. Bilinguals who code-switch differ in their habits of using their languages. In a dense code-switching environment, bilinguals may interact in a context in which they switch languages between turns and sentences, or switch languages within the same utterance and tend to adapt words from one language to fit within the structure of the other (e.g., Green, 2011). They may also differ in the frequency and daily use of code-switching (Rodriguez-Fornells et al., 2011). The adaptive control hypothesis (Green and Abutalebi, 2013; Green and Wei, 2014) assumes that task schemas do not compete in a dense code-switching context, but cooperate to tolerate alternative forms depending on how much they fit within the given context. However, when dense code-switching is not a common language practice of the bilinguals between a pair of languages that they speak, it is likely that encountering a lexical switch within the same utterance is unexpected and imposes higher cognitive processing demands. Difference also exist among the Algerian bilinguals who interact in AA-FR dense code-switching: there are those who are heavy code-switchers (frequently code-switch) and others who are light code-switchers (not frequently code-switch). However, all these bilinguals do share the fact that they do not use Standard Arabic in everyday interactions and thus, SA-FR code-switching is not common practice for all of them. We expected larger effect of frequency of code-switching on language switch expectancy for light code-switchers than for heavy code-switchers. In particular, the difference between light and heavy switchers should be seen more in the AA-FR switches.

Finally, the study also sought to examine whether language expectancy in switching is modulated by French language proficiency. Proficiency may affect language activation in bilinguals with higher proficiency bilinguals showing more parallel activation than lower proficiency bilinguals (Blumenfeld and Marian, 2013), or showing a better control for L1 interference (Elston-Güttler et al., 2005). Proficient bilinguals may not need inhibition to produce words in one language only (e.g., Costa and Santesteban, 2004; Costa et al., 2006). Gollan and Ferreira (2009) noticed that balanced Spanish–English bilinguals switched languages more often than unbalanced bilinguals when they voluntarily switched languages in a naming task. The writers concluded that voluntary and cued language mixing became easier as proficiency increased because the more proficient bilinguals did not need to inhibit their dominant language in order to make the other language as much accessible. Language proficiency also affected accuracy rates in naming studies (e.g., Schwartz and Kroll, 2006) in which less proficient bilinguals had significantly higher naming error rates than the highly proficient bilinguals. In addition, proficiency affected the ability of the bilingual to stay in one language as needed and was seen to affect the word category that is most vulnerable to slips of the tongue (Poulisse, 2000), but also to affect the type of constituents in sentential code-switching among bilingual speakers (Backus, 1996; Myers-Scotton, 2006). Finally, language proficiency was found to interfere with the effect of semantic constraint. Bilinguals with more proficiency in L2 showed reduced cognate facilitation in high-constraint sentences (e.g., Libben and Titone, 2009). The goal in the current study was therefore to see whether high proficient speakers of French differ from low proficient bilingual speakers of French in the expectancy of a language switch during listening to Algerian Arabic and Standard Arabic base languages.

To summarize, the research questions addressed in the current study are: (1) Is language expectancy in code-switching dependent on the base language? That is, does language expectancy differ between a typical code-switching (AA-FR) and a non-typical code-switching (SA-FR) context? (2) Do semantic constraints affect language expectancy in code-switching? (3) Is language expectancy dependent on the frequency of code-switching? (4) Does French L2 proficiency modulate the expectancy of language switching?

To answer these questions, we measured reaction times to the French NP code-switches using a CMN task (e.g., Hernández et al., 1996; Love et al., 2003). The CMN is an on-line method that is sensitive to sentential and lexical priming. In this task, the participants listen to a sentence, and at a particular point the sentence stops for a moment and a target word appears visually in the center of the computer screen. While the participants name the target word as fast and accurately as possible their reaction times are recorded. This task is similar to the Cross-Modal Lexical Priming task (CMLP; e.g., Li, 1996; Heredia and Blumentritt, 2002) with one critical difference. In the CMLP the flow of the sentence is not interrupted which makes it unlikely for the listener to engage in strategic processing (Heredia and Stewart, 2002). Thus, similarly to the CMLP task, the auditory presentation of the stimuli in the CMN used in the current study was not interrupted, and the visual target words appeared on the screen following the natural flow of the sentence without a time interval. The use of this task in this study allows us to obtain reaction times that reflect the processing of the context immediately as the sentence unfolds. Because the target words are presented at the offset of the last word that was heard, the effects observed on the participants’ reaction times can be attributed to the participants’ analysis of the sentence attained at the end of the auditory fragments.

The CMN has been demonstrated to measure what is active at certain moments in time during continuing processing of a sentence (e.g., Love et al., 2003). Its sensitivity to semantic as well as contextual effects have been well-documented (e.g., Tabossi, 1988, 1996; Hernández et al., 1996; Love et al., 2003). In particular, results from studies on code-switched sentences using CMN have shown that the paradigm is sensitive to sentential context (e.g., Hernández et al., 1996; Cieślicka and Heredia, 2015). In contrast to Hernández et al. (1996), we presented sentences in a mixed rather than a blocked fashion because semantic and lexical facilitation in Hernández et al. (1996) occurred in the blocked condition when the bilinguals knew what language to expect. However, with a mixed block condition, it is unlikely that participants develop strategic responses since they are not aware of what comes next. In the latter case, any difference observed between the conditions would be the product of sentential effects. In addition, priming effect in these studies depended on the language manipulation and was larger for the language of everyday interaction. Along with the advantages mentioned above, CMN is a good method to use in the current study for methodological reasons. The auditory presentation of the stimuli eliminates the issue of presenting Algerian Arabic, traditionally spoken only, in written script. Listening to the stimuli also avoid the visual appearance of both languages in the same sentence which is mostly stigmatized and considered ungrammatical by many bilinguals even those who code-switch. Since CMN is sensitive to what is active at points in time during the ongoing processing, it can test whether French is activated and available after listening to Algerian Arabic more than after listening to Standard Arabic.

Two factors were manipulated in this study: base language, that is, the language preceding the target word (Algerian Arabic or Standard Arabic) and semantic constraint of the context preceding the target word (High or low constrained contexts). The results in this study extend previous findings by exploring the effect of language use and frequency of code-switching. Algerian bilinguals listened to fragments of sentences either in Algerian Arabic or in Standard Arabic then immediately after named a target NP that was always in French. The target NP was thus heard in four different switching conditions: Algerian Arabic high-constraint context (AAH), Algerian Arabic low-constraint context (AAL), Standard Arabic high-constraint context (SAH), and Standard Arabic low-constraint context (SAL). Since all critical trials are code-switching trials, faster reaction times to the presented target words can be interpreted as ease of processing due to language switching expectation. Reaction times to French switches are compared in both base languages (AA and SA) and in both semantic constraint contexts (high and low). The following hypotheses and predictions are formed based on the research questions.

Concerning the first question, if the habit of switching between a certain pair of languages affects the expectancy of a language switching, there should be a base language effect. Participants should expect a switch to French when the base language is Algerian Arabic but not when the base language is Standard Arabic. This is because a switch to French is not typically expected when listening to Standard Arabic and because AA-FR code-switching is the default language switching in everyday conversation. Reaction times to French switches should be faster when Algerian Arabic is the base language than when Standard Arabic is the base language.

As regard to the second research question, if semantic context affects the expectation of a language switch, it is predicted that reaction times to switches in the high-constraint context should be faster than in the low-constraint context, and in particular after Algerian Arabic base language than after Standard Arabic base language. This is because the highly constraining context provides more semantic clues that help in predicting upcoming words, and previous studies showed that more predictable words are processed faster in naming (e.g., McClelland and O’Regan, 1981; Stanovich and West, 1981). In addition, expectations for a French continuation in a Standard Arabic context is weaker than in an Algerian Arabic context.

For the third research question, if language switch expectancy depends on the bilingual’s recurrent switching habits, then reaction times for heavy code-switchers (those who frequently code-switch) should differ from light code-switchers (those who do not switch frequently). In addition, if heavy code-switchers are more experienced with dense code-switching contexts they should employ their cooperative control processes more than light code-switchers. In particular, we should see a difference in processing a switch depending on the extent of daily code-switching. In other words, bilinguals who code-switch more frequently should show more cooperative processes, which will be reflected in their reaction times. We also predict switching habits to interact with base language effect. Because AA-FR code-switching is more recurrent, anticipation of a language switch is more likely when Algerian Arabic is the base language. The difference between heavy code-switchers and light code-switchers should therefore be more apparent in AA-FR than in SA-FR code-switching. We finally predict switching habits to interact with semantic constraint. If language tasks schemas are in more cooperative mode for the heavy code-switchers compared to light code-switchers, then the effect of semantic constraint should differ between heavy and light code-switchers and more so in Algerian Arabic than in Standard Arabic base language.

For the last question, if French proficiency modulates the expectancy of language switching, then high proficiency bilinguals should be different from low proficiency bilinguals in processing the switch. If high proficiency bilinguals show more parallel activation for French, it is predicted that they should be faster overall than low proficient bilinguals and they should show a smaller effect for base language than low proficient bilinguals. Proficiency in French may also modulate the effect of semantic constraint. Highly proficient bilinguals in Libben and Titone (2009) showed reduced cognate facilitation in the high-constraint context which let them conclude that proficiency with semantic constraint can support language selectivity during the early stages of lexical access. If this is the case, high proficient bilinguals may show smaller semantic constraint effects than low proficient bilinguals.

## Materials and Methods

### Ethical Approval

The current study was approved by the University of Florida Institutional Review Board (IRB) 02: Protocol #2014-U-0904.

### Participants

Sixty-five Algerian college students mostly from the National School of Computer Science and the National School of Polytechnics in Algiers participated in this experiment (mean age 22, range 18–25; 31 female and 34 male). All participants were either born in Algiers or came to Algiers at an early age. They all had Algerian Arabic as their mother tongue and either started learning Standard Arabic when they started school or at kindergarten or mosque. However, participants differed in the time of acquiring French. Early bilinguals reported that they started French together with Algerian Arabic or shortly after. They also said that they watched cartoons mostly in French. Late bilinguals started French at school at around age 8, and may or may not have watched cartoons in French before they started French at school. All Bilinguals also claimed that they code-switch with friends and family, but they differ in the time when they started code-switching or in how often they code-switch. Participants were recruited by means of an announcement for the study via leaflets containing conditions for participation and were paid for their participation.

#### Language Proficiency Assessment

To assess French proficiency, participants completed the French Cloze test developed by Tremblay (2011). The test consists of a text that contains blanks (deleted words) and which the participants had to read and fill in each blank with one word. Of the 45 blanks, 23 were content words (e.g., nouns, main verbs, adjectives, etc.) and 22 were function words (determiners, pronouns, prepositions, etc.). Standard Arabic proficiency was assessed using a Cloze test developed for the purpose of this study. The test contains 35 deleted words of which 26 were content words and 9 were function words. In order to standardize the Arabic Cloze test, 12 Algerian speakers who did not take part in the actual study, completed the test. From their responses, a bank of acceptable answers was created and used for scoring the test. The scores from both tests were converted into percent accuracy rates.

#### Language History and Switching Habits Questionnaire

The participants completed a language questionnaire. This was a French translated version of “*The assessment of code-switching experience survey*” (ACSES) developed by Blackburn and Wicha (2011). The questionnaire starts with some autobiographical questions concerning the participants’ age, gender, place of birth, and residence. The participants also self-rated their proficiency in reading, writing, speaking, and listening for Algerian Arabic, Standard Arabic, and French. The questionnaire included multiple choice questions concerning the participants’ daily use of languages, their code-switching habits, reasons for code-switching and their attitudes toward code-switching. Code-switching scores are the averages of daily use of languages and frequency of code-switching.

In addition to these tests participants were given a semantic fluency test, the Simon task, a working memory test, and an interview. These data will not be reported here.

### Material and Design

The stimuli contained a total of 32 non-cognate French target words (underscored in **Table 1**). We used non-cognate nouns because it would be hard to find cognate nouns that are shared between Algerian Arabic, Standard Arabic, and French. Cognates are typically loans from French into Algerian Arabic but not into Standard Arabic. The French words were embedded in high and low-constraint Algerian Arabic and Standard Arabic sentences. Stimuli, thus, included 16 AA-FR code-switched sentences and 16 SA-FR code-switched sentences. The cloze probability of the sentences was determined on the basis of a web-based completion study on 76 Algerian bilinguals not participating in the actual study. The sentences were initially presented in French leaving the final noun phrase out for the participants to complete with three possible best completions. The mean cloze probabilities for the target words in the high-constraint sentences was (0.77), and in the low-constraint sentences was (0.06). Sentences in Algerian Arabic and those in Standard Arabic were close translations to the French sentences as reviewed by three Algerian bilingual speakers. In all sentences, the target words were French noun phrases formed with a feminine noun and a feminine definite article that appeared at the end of the sentence. Masculine nouns were avoided because the French masculine article *le* preceding the noun is mostly replaced by the Algerian Arabic article *∂l* which needs to be assimilated to the initial consonant of the noun when it is a *solar,* i.e., a coronal consonant. Noun phrases in this study were visually presented in French. Thus, in the case when the context constrains toward a masculine noun that starts with a coronal sound, participants may anticipate the assimilated article ə*l* but find the unassimilated article *le* instead. This might incur extra processing that confounds with the processing that is due to language switch expectation in the controlled conditions. The mean length of sentences was (mean = 8, range = 6–11 words) and in milliseconds (mean = 3071 ms, range = 1756–4705 ms). Experimental target nouns were controlled for length and frequency for the purpose of Latin Square (see below): length (mean = 8, range = 6–11 characters) and frequency (mean = 6, range = 4–8). The frequency of the French nouns was based on an online survey completed by twelve Algerian bilinguals. A sample sentence in the four conditions is displayed in **Table 1**.

**Table 1:** Sample of experimental item set.

Condition	Sample sentence
AA base language High-constraint (AAH)	Kul-ma naɣslu ssnaan lazem nʃallu **la bouche.** “Every time we wash the teeth we rinse the mouth.”
SA base language High-constraint (SAH)	fi kuli maratin naɣsilu fiha ʔal ʔasnaan jadʒibu ʔan naʃSt^ʕ^ifa **la bouche.** “Every time we wash the teeth we rinse the mouth.”
AA base language Low-constraint (AAL)	Had l’ewled ma rqadsh 3laxat^ʕ^er∫ kan ʕendu s^ʕ^t^ʕ^ar fi **la bouche.** “This boy did not sleep because he had pain in the mouth.”
SA base language Low-constraint (SAL)	ʔina haað ʔal walad lam janam liʔanahu kaana juʕaani min ʔalamin fi **la bouche.** “This boy did not sleep because he had pain in the mouth.”


Another 64 sentences were constructed as fillers. Half of the fillers used Algerian Arabic and half used Standard Arabic. To avoid the adoption of a strategy by the participants, half of the fillers had switches at different points of the sentences. Filler switches appeared either earlier toward the beginning of the sentences, in the middle or toward the end of the sentences but never word finally. The other half did not contain switches and were, therefore, only heard. The filler switches were either nouns, verbs, adjectives or adverbs. All experimental and filler sentences contained switching from Arabic to French because this type is more common than switching from French to Arabic. In addition, Algerian Arabic is not traditionally written, rendering Algerian Arabic language unsuitable for the targets in the present paradigm. Four lists of stimuli were constructed using Latin Square, such that each list contained one sentence in each of the four conditions, and no list contained more than one version of each sentence. Each participant saw only one list of 96 sentences and each experimental target NP appeared only once in each list. The fillers were the same across the four lists. Sentences in each list were pseudo-randomized to avoid order effect, and lists were randomly assigned to participants. All sentences were recorded by the same bilingual Algerian female speaker in a soundproof boot using a Marantz PMD660 digital recorder, recording 16-bit stereo PCM sound at a sampling rate of 44.1 kHz. In order to minimize co-articulation, a dummy word “*huda*” was inserted instead of the actual target words. The auditory sentences were coded and segmented using (Boersma, 2001): the speech signal was cut just before the presentation of the target word, using nearest zero crossing selection, and the durations of the speech segments were extracted. The auditory sentences were then normalized to minimize the difference in amplitude across all sentences.

### Procedure

Once arrived to the study site, the participants first completed the French proficiency cloze test, then completed the following tests not included in the analysis of the current study: a French semantic fluency test, the Simon task, the memory test and the interview in this order. After a short microphone test, participants started the CMN experiment with a practice session. The practice task consisted of five sample trials resembling the experimental and filler sentences, that is, the target words appeared at the end of a sentence, somewhere in the middle or the trial did not have any visual target words. During the practice session the experimenter remained next to the participants and gave feedback on their performance. When the participants started the experiment, the experimenter remained in the room but withdrew to a corner. After the completion of the naming experiment, the participants completed the cloze test for Standard Arabic proficiency followed by the Arabic semantic fluency task (not included in this analysis), and the language history and switching habits questionnaire.

Stimuli in the CMN task were presented on the screen of a laptop computer using the E-Prime 2.0 software (Psychology Software Tools, Pittsburgh, PA, USA). Participants were seated at about 50 cm from the computer with a microphone on a stand sitting in between, and a response button box on the right side of the computer. Participants also wore a headphone set with a microphone attached to the computer digital array mic. While the headphone presented the auditory stimuli, the head-mounted microphone recorded the participants’ naming responses. Reaction times to targets naming were collected using a voice trigger via the standing microphone attached to the response button box. The participants were instructed to listen carefully to the content and read the words that appeared on the screen as quickly and accurately as possible. To make sure the participants paid attention to the content, they were told they would be asked some questions at the end. Participants had to press any button on the response box to proceed to a trial. First, the participant saw a *Prêt* “Ready” sign at the center of the screen and pressed a button to proceed when they were ready to start. The ready sign then changed to a cross sign “+” inside a rectangle and remained on the screen while the participants listened to the auditory part of the sentence. At the offset of the last word, the cross sign disappeared and the target word was displayed instead for a duration that was equal to the word length (in milliseconds) times 150 plus 1200 ms. The participants were asked to read the words out loud. When the display time ended the “Ready” sign started the following trial in the case of an experimental trial. In the case of a filler trial, in which the naming occurred somewhere in the middle of the sentence, the cross sign was displayed again and the participants heard the final auditory part that completed the sentence before they saw the “Ready” sign again. When the fillers did not contain a target word to read, participants heard the entire sentence until they saw the “Ready” sign to proceed to the next trial.

## Results

The analysis was conducted on correct responses only. A correct response is one which was clearly fluent with no stammering or hesitation. Correct responses after self-correction were not accepted. Answers which were ambiguous due to unclear pronunciation or lack of audibility were presented to another Algerian-French bilingual speaker. If the bilingual speaker could not identify the word or hesitated about identifying it, that word was excluded from the analysis. Raw data consisted of 2080 data points. Incorrect or non-identifiable responses constituted 1.3% (27 data points). Accurate responses were equally distributed across the four conditions: Algerian Arabic high-constraint: AAH (98%); Standard Arabic high-constraint: SAH (99%), Algerian Arabic low-constraint: AAL (99%); Standard Arabic low-constraint: SAL (98%). Overall accurate data was 2053 data points (98.7%). We conducted all analyses on the log transformed residual reaction times in order to control for potential effects of target word length, frequency and the position of the trial in the experiment. Log transformed reaction times were residualized for length (in characters) and frequency of the target words, and for the trial number, that is, the order in which a certain item appeared in the experiment. Residuals were calculated by means of a linear mixed effect model conducted on the log transformed response times in the experimental trials, with target word length (in number of characters), target word frequency, and trial order as fixed effects, and a by-subject random intercept. Response times estimated by this model were then subtracted from the log transformed response times to obtain the log residual response times. Outliers were then removed from each condition for each participant using the mean ± 3 standard deviation method. After cutting off for outliers, 2028 data points (98.8%) remained that is 25 data points (1.2%) were removed.

In order to determine which factors to include in the model, tests of correlations using the rcorr () function in the Hmisc package were utilized in order to further explore the correlations/covariances and significance levels for Pearson and Spearman correlations between code-switching habits (as measured by the ACSES questionnaire), age of acquisition of French and proficiency in French (as determined by the Cloze test). There was a medium-sized negative correlation between Age of acquisition and proficiency, *r* = -0.36; *p* < 0.01. The later French was acquired, the less proficient the bilingual. However, correlations between code-switching and age of acquisition, and code-switching and proficiency did not reach significance: with age of acquisition *r* = 0.09; *p* = 0.49; with proficiency, *r* = -0.09; *p* = 0.47. Based on these correlation results, proficiency but not age of acquisition was included as a factor in the analysis of the target word naming times.

Naming latencies to the French target words were then analyzed using a linear mixed effects model lmer in R (version 3.1.3, R Core Team, 2015) as implemented in the package lme4 (version 1.1-7, Bates et al., 2015). The model included semantic constraint (High Cloze/Low Cloze, with “high cloze” coded as -0.5 and “low cloze” as 0.5), base language (AA/SA, with “AA” coded as -0.5 and “SA” as 0.5), and the continuous variables French Proficiency (FrProf) and code-switching habits (CS) as fixed effects. In addition, the interactions between each two of these factors (except that between proficiency and code switching) were also included as fixed effects. The analysis contained a random effect structure which included by-subject and by-item random intercepts, with the fixed effects constraint, base language and their interactions as by-subject and by-item random slopes. The fixed effects were group-mean centered to minimize collinearity. After centering, the maximal variance inflation factor was 1.04, and there were no signs of collinearity in the analysis (fixed effect correlations *r*s < 0.2). Significance of the fixed effects was obtained on the basis of the *t*-values of the estimates of the coefficients. Absolute *t*-values of 1.96 or larger were considered significant. To explore significant interactions produced by the model, we followed-up with separate linear mixed-effects models fitted for each specific group. In addition, we calculated effect sizes “Cohen’s *d*” following Dunlap et al. (1996) to compare groups on the basis of the magnitude of a statistically significant effect and we provided 95% confidence interval (**Table 5**). Note that the analysis was conducted on the log transformed residualized reaction times, however, the means in the main text are given for the raw reaction times for the reader’s convenience.

### Overall Analysis

A maximal model was fitted and the model converged without simplifying the random slope structure. Analysis on the residual RTs for the entire group of participants as summarized in **Table 2** shows a main effect for semantic constraint, a semantic constraint by code-switching habits interaction, and a semantic constraint by proficiency interaction. However, base language effect was not significant and no interaction with base language was significant. We will elaborate on each of these effects below in the light of the research questions.

**Table 2:** Results of the residual naming latencies mixed effects analysis for whole group.

Effect	β	*SE*	*t*-value
Intercept (mean)	-0.006	0.007	-0.83
Semantic constraint	0.03	0.01	2.78*
Base language	0.01	0.01	1.02
French proficiency	0.0005	0.0005	1.13
Code-switching	0.004	0.005	0.89
Semantic constraint^∗^Base language	-0.02	0.02	-0.93
Semantic constraint^∗^Code-switching	-0.02	0.01	-1.98*
Semantic constraint^∗^French proficiency	-0.002	0.001	-2.64*
Base language^∗^Code-switching	-0.01	0.01	-1.64
Base language^∗^French proficiency	0.0003	0.001	0.28


*SE, standard error;*^∗^*significant t*-*value* (*p* < 0.05).

#### Language Switch Expectancy and Semantic Constraints

There was a significant main effect of semantic constraint (**Table 2**). Participants’ naming latencies were shorter in the high-constraint sentences (*M* = 565 ms, *SD* = 158) than in the low-constraint sentences (*M* = 583 ms, *SD* = 179), suggesting that it was easier for the participants to anticipate the target words more when the preceding context provided rich semantic clues about the forthcoming lexical items.

**FIGURE 1:**
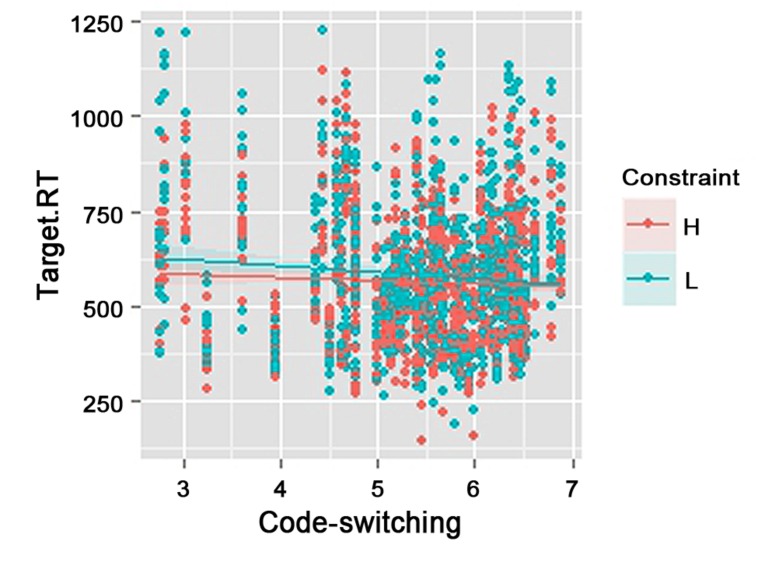
**Effect of semantic constraint (H: High, L: Low) and code-switching habits (Scores based on switching questionnaire: 7 = highest score) on language expectancy (Target.RT: naming latencies to French switches in milliseconds)**.

The interaction between semantic constraint and code-switching habits was significant (**Table 2**). As shown in **Figure 1**, the semantic effect (faster naming latencies for high-constraint than low-constraint sentences) was larger for the participants who code-switched less frequently than for those who code-switched more frequently.

**FIGURE 2:**
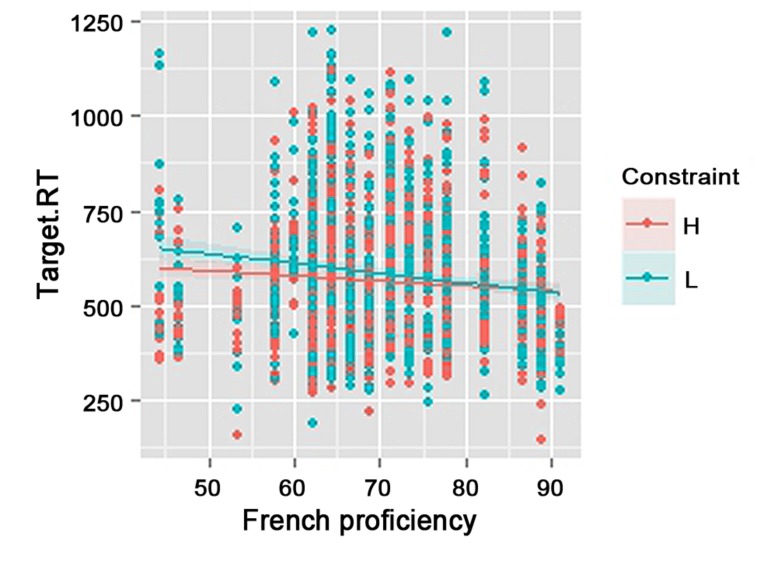
**Effect of semantic constraint (H: High, L: Low) and French proficiency (Scores based on French Cloze test) on language expectancy (Target.RT: naming latencies to French switches in milliseconds)**.

Semantic constraint also interacted with French proficiency (**Table 2**) revealing a significant difference between high proficiency bilinguals and low proficiency bilinguals in the effect of semantic constraint of a sentence context. Overall, highly proficient participants were faster and showed reduced semantic constraint effect than low proficient participants (**Figure 2**), suggesting that bilingual code-switchers who are more proficient in L2 French tend to expect switching into French more than code-switchers of lower L2 proficiency.

### Exploring the Interactions

#### Semantic Constraints and Code-Switching Habits

In order to further explore semantic constraint by code-switching habits interaction, we examined the bilinguals at the two extremities of the continuous code-switching line. We compared bilinguals with the lowest code-switching scores to bilinguals with the highest code-switching scores in the overall group. Twenty participants in each code-switching group type (light code-switchers/heavy code-switchers) were selected based on their code-switching scores in the language history and switching habits questionnaire (ACSES). The code-switching score was calculated based on the averages between daily use of languages and code-switching habits. The code-switching groups were matched on age, language proficiency and age of acquisition. **Table 3** reports the characteristics of the participants in the code-switching groups.

**Table 3:** Participant characteristics in the code-switching groups.

Characteristics	Code-switching group		
	Light code-switchers CS ≤ 5.18, *n* = 20	Heavy code-switchers CS ≥ 6, *n* = 20	*p*-value
Code-switching	4.29	6.37	0.000*
Age	22.05	22.00	0.90
French proficiency	73.44	71.44	0.53
Age of FR acquisition	6.30	7.15	0.21
Standard Arabic proficiency	74.55	76.76	0.54
Age of SA acquisition	5.10	5.15	0.81


^∗^Significant *p*-value < 0.05.

Treating code-switching as categorical, we constructed a linear mixed effect lmer for the heavy code-switchers and a separate lmer for the light code-switchers. Both lmer models contained constraint (High Cloze/Low Cloze, with “high cloze” coded as -0.5 and “low cloze” as 0.5), base language (AA/SA, with “AA” coded as -0.5 and “SA” as 0.5), the continuous variable French Proficiency (FrProf) and the interactions between each two of these factors as fixed effects. The random effects structure was similar to that in the overall analysis model. As before, the fixed effects were centered to minimize collinearity. After centering, the maximal variance inflation factor was smaller than 1.05, and there were no signs of collinearity in the analysis (fixed effect correlations *r*s < 0.2).

The analysis from both lmer models for the code-switching groups separately showed a significant main effect of semantic constraint in the light code-switching group: [β: 0.04, *SE*: 0.01, *t*: 2.48], but not in the heavy code-switching group [β: 0.01, *SE*: 0.02, *t*: 0.81]. Comparison of the effect sizes confirmed that the light-code switchers showed a larger effect of semantic constraint (Cohen’s *d*: 0.59) than the heavy code-switchers (0.17). The difference between high and low constraints was larger for the light code-switchers (naming took 24 ms longer in the low than the high constrained context) than for the heavy code-switchers (10 ms). In particular, the means of naming latencies in high and low-constraint contexts revealed that light code-switchers differed from heavy code-switchers in the highly constraining context. Light code-switchers had shorter naming latencies (*M* = 576 ms, *SD* = 184) than heavy code-switchers (*M* = 595 ms, *SD* = 149) indicating that they processed the switch faster. In the low constraining context, naming latencies in light code-switchers (*M* = 600 ms, *SD* = 209) were not different from those in heavy code-switchers (*M* = 605 ms, *SD* = 170). This may also suggest that bilinguals in the light code-switching group anticipated a language switch compared to bilinguals in the heavy code-switching group.

#### Semantic Constraints and French Proficiency

The interaction between semantic constraint and proficiency was explored by comparing the effect of semantic constraint in the lowest proficient bilinguals and the highest proficient bilinguals from the overall group. Based on scores in the French proficiency test, two groups (low proficient/high proficient) were selected, each containing 20 participants. The high proficiency group started learning French at an earlier age than the low proficiency group (**Table 4**), but the two proficiency groups were matched on age, code-switching habits, proficiency in Standard Arabic and age of Acquisition of Arabic.

**Table 4:** Participant characteristics in the proficiency groups.

Characteristics	Proficiency group		
	Low proficient Accuracy < 65%, *n* = 20	High proficient Accuracy ≥ 77%, *n* = 20	*p*-value
Code-switching score	5.36	5.24	0.72
Age	22.05	21.60	0.34
French proficiency	60.11	82.89	0.000*
Age of FR acquisition	7.50	5.40	0.001*
Standard Arabic proficiency	73.38	75.44	0.56
Age of SA acquisition	4.85	5.05	0.40


^∗^Significant difference between the two groups (*p*-value < 0.05).

**Table 5:** Ninety five percentage confidence interval of the mean differences in raw RTs for semantic effect in overall and group analyses.

Semantic effect by group	95% confidence interval		Semantic effect by base language	95% confidence interval	
	2.5	97.5		2.5	97.5
Overall	-32.79	-3.32	Overall AA	-47.57	-10.62
Light code-switching	-55.77	-6.88	Overall SA	-29.39	4.37
Heavy code-switching	-37.52	15.33	Light code-switching AA	-80.58	-4.97
Low proficiency	-71.53	-11.73	Light code-switching SA	-52.59	13.35
High proficiency	-24.98	5.13	Low proficiency AA	-104.40	-4.143
			Low proficiency SA	-68.96	16.96


We constructed two linear mixed effects lmer models separately for the two proficiency groups with proficiency treated as categorical to examine sematic effect significance. The models contained constraint (High Cloze/Low Cloze, with “high cloze” coded as -0.5 and “low cloze” as 0.5), base language (AA/SA, with “AA” coded as -0.5 and “SA” as 0.5), code-switching habits (CS) as a continuous variable, and the interactions between each two of these factors as fixed effects. The random effects structure was similar to that in the overall analysis model. The fixed effects were centered to minimize collinearity. The maximal variance inflation factor after centering was smaller than 1.04, and there were no signs of collinearity in the analysis (fixed effect correlations *r*s < 0.2).

The effect of semantic constraint was still significant in the low proficiency group: [β: 0.06, *SE*: 0.02, *t*: 2.92]. Naming latencies in the low proficient bilinguals were 32 ms shorter in the high-constraint context (*M* = 571, *SD* = 172) compared to the low-constraint context (*M* = 603 ms, *SD* = 208). However, the effect of semantic constraint in the high proficiency group was not statistically significant: [β: 0.02, *SE*: 0.02, *t*: 1.08]. High proficiency bilinguals were only 10 ms faster in responding to the targets in the high (*M* = 523, *SD* = 135) than in the low (*M* = 533, *SD* = 145) constraint contexts. Effect sizes confirmed these results: there was a larger effect size in the low proficiency group (0.7) but a relatively small effect size in the high proficiency group (0.31).

#### Effects of Base Language

Although the analysis does not show an interaction between semantic constraint and base language we wanted to explore the effect of semantic constraint in each base language separately given that we hypothesized that semantic constraint effect should be more visible in Algerian Arabic because of the high expectation of a French continuation in daily language use. Separate lmer models for Algerian Arabic base language trials and Standard Arabic base language trials revealed a significant main effect of semantic constraint in Algerian Arabic base language: [β: 0.04, *SE*: 0.01, *t*: 2.53], but not in the Standard Arabic base language: [β: 0.02, *SE:* 0.01, *t*: 1.635]. Naming latencies to the French targets were shorter in the high semantically constraining sentences (*M* = 558 ms, *SD* = 150) than in the low semantically constraining sentences (*M* = 584 ms, *SD* = 184) when the base language was Algerian Arabic. However, when the base language was Standard Arabic naming latencies in the high constraint context were not significantly faster (*M* = 571 ms, *SD* = 166) than those in the low constraint context (*M* = 581 ms, *SD* = 174). The results suggest that participants benefited more from semantic manipulation by using the semantic cues in the high-constraint sentences during listening to Algerian Arabic comparted to listening to Standard Arabic. The effect sizes confirmed this interpretation. Although effect sizes are both relatively small Cohen’s *d* was smaller in the Standard Arabic base language (0.18) than it was in Algerian Arabic base language trials (0.36).

The effect of semantic constraint in the code-switching groups was found to be larger for the light code-switchers. We conducted separate analyses by base language in order to see in which base language was the effect size more important. Comparison of the analyses for Algerian Arabic base language trials and Standard Arabic base language trials in light code-switchers showed a significant main effect of semantic constraint in Algerian Arabic base language: [β: 0.05, *SE*: 0.02, *t*: 2.28], mean naming latencies in the high-constraint context (*M* = 562; *SD* = 163), and in the low-constraint context (*M* = 596; *SD* = 209), but not in Standard Arabic base language: [β: 0.03, *SE*: 0.02, *t*: 1.13], mean naming latencies in the high-constraint context (*M =* 591; *SD* = 202), and in the low-constraint context (*M* = 604; *SD* = 211). Comparison of the effect sizes confirmed these results: Cohen’s *d* was (0.46) in Algerian Arabic base language but it was (0.27) in Standard Arabic base language. Once again, the results suggest that the effect of semantic constraint was driven by the context language that is commonly used in interactional contexts and is part of the more typical AA-FR code-switching.

Similarly, the effect of semantic constraint was larger in the low proficiency group. Analysis by base language in the low proficiency group revealed a significant semantic constraint effect in Algerian Arabic base language trials: [β: 0.08, *SE*: 0.03, *t*: 2.25], mean naming latencies in the high-constraint context (*M* = 564; *SD* = 163) were 45 ms shorter than those in the low-constraint context (*M =* 609; *SD* = 219). However, semantic constraint effect was not significant in Standard Arabic base language trials: [β: 0.04, *SE*: 0.03, *t*: 1.21], mean naming latencies in the high-constraint context (*M* = 578; *SD* = 181) were 19 ms shorter than naming latencies in the low-constraint context (*M* = 597; *SD* = 196). Comparison of the effect sizes showed a larger effect size in Algerian Arabic base language (0.48) than in Standard Arabic base language (0.26).

These results are in line with our prediction that the difference in semantic constraint effect should be seen more in the typical AA-FR code-switching context than in the atypical SA-FR code-switching context. The fact that naming latencies were constantly shorter and the effect sizes constantly larger in Algerian Arabic than in Standard Arabic is evidence that base language did affect the processing of the switch. Semantic facilitation in the high-constraint context in Algerian Arabic base language promoted the processing of a code-switch. In particular, the results suggest that when the switch is part of the typical language pair that is repeatedly used in conversation, its processing is easier. It may also suggest that participants could anticipate a language switch when they heard Algerian Arabic. An observation worthy of notice is that Algerian Arabic and standard Arabic are rather similar in several aspects, and thus differences in effect sizes should not be expected. In this respect, the observed differences between Algerian Arabic and standard Arabic base languages in the different groups, though not always large, are informative for models of code switching.

## Discussion

In this study, we sought to examine the effect of language use and semantic constraints on the expectancy of a language switch during listening comprehension in Algerian bilingual speakers. In particular, expectation of a language switch was compared between two types of code-switched sentences that involved different pairs of languages/varieties. The first occurs between Algerian Arabic and French and is typically conversational and frequent among the bilingual community that code-switches. The second type involves code-switching between Standard Arabic and French which is neither interactional nor typical of Algerian bilinguals. Participants heard the first part of the code-switched sentences presented either in Algerian Arabic or Standard Arabic then, immediately after, read a French NP that completed the first parts. Naming latencies to the French NPs were measured and compared. Faster reaction times suggested an easier processing of the target word, which can be interpreted as a higher expectation of a language switch and ease of switch processing. We asked (1) whether language expectancy in code-switching depends on the base language; (2) whether semantic constraints affect language expectancy in code-switching; (3) whether language expectancy is dependent on the frequency of code-switching; and (4) whether French L2 proficiency modulates the expectancy of language switching.

The findings revealed three effects: semantic constraint effect; an interaction between constraint and code-switching habits; and an interaction between constraint and French proficiency. Bilinguals were significantly faster in the high than in the low-constraint context, suggesting that a language switch is more expected and/or the switch is easier to process when it is supported by the semantic information of the sentence context. This also suggests that the CMN task was sensitive to sentence context and to lexical activation. In addition, the semantic constraint effect, that is, the difference between reaction times in high and low-constraint contexts, was larger when the base language was Algerian Arabic than when it was Standard Arabic. This suggests that the listeners made more use of the semantic cues provided by the high-constraint context in the more typical code-switching that is more recurrent in the everyday interactions. In addition, the frequency of daily code switching modulated the effect of semantic constraint of a sentence context. Light code-switchers but not heavy code-switchers were significantly faster in the high-constraint context than in the low-constraint context preceding the switch. This suggests that the habit of switching between languages interferes with our predictions and with the state of activation of both languages. However, these results look counterintuitive. One would assume that the more a bilingual code-switches the more he/she expects a language switch. We will provide a speculative interpretation of this below. Finally, we found that high proficiency bilinguals had shorter naming latencies than low proficiency bilinguals. French proficiency modulated the effect of semantic constraint on language switch expectancy. Bilinguals with low proficiency in French showed larger constraint effect, with faster reaction times in high-compared to low-constraint context. As proficiency increased the difference in naming latencies between high and low-constraint contexts became smaller, probably due to the overall increase in speed, leading to a reduced effect of sentence context.

### Theoretical Accounts and Implications

The major finding of the current study is that the effect of semantic context is contingent on the bilingual’s language use. In particular, the effect of semantic context occurred to the extent to which the bilinguals code-switch in everyday interactions. Semantic constraint effects were reduced in bilinguals who frequently code-switch, but were visible in bilinguals who code-switch less frequently. Studies reporting reduced sentence influence in the high-constraint context (e.g., Altarriba et al., 1996; Titone et al., 2011) suggested that the readers in the highly constrained context generate semantic as well as lexical features of the upcoming words in the context language. However, when the target words mismatch the expected words in phonology, there is extra processing. They also suggested that the semantic context can selectively activate a word in one language. Titone et al. (2011) noted that L1 can activate L2 in a highly constraining context when L2 phonology is salient (e.g., when sentences from L1 and L2 are intermixed in the study), and when the bilinguals are highly proficient in their L2. In a recent study, Boukadi et al. (2015) tested Tunisian Arabic- French bilinguals who are moderately proficient in French. The study used picture-word interference task in monolingual and bilingual contexts. When the context was monolingual, naming latencies were affected only by phonological facilitation, suggesting that lexical selection proceeded in a selective manner. In the bilingual context, a phono-translation effect as well as phonological and semantic effects were found suggesting that lexical selection is non-specific. The writers suggest that the bilingual lexical selection is dynamic and depends on factors such as the experimental language context (monolingual or bilingual). The degree of activation of both languages determines whether lexical selection functions in a language non-specific or in a language-specific manner. If the extra processing in the heavy code-switching group is due to the fact that the participants generated lexical features of the upcoming words in the context language, then the question is why participants in the light code-switching group did not generate lexical features in the context language. If on the other hand, facilitation in the high-constraint context in the light code-switching group is attributed to the activation of L2, then the question is why the heavy code-switchers did not activate L2 to the same extent as the light code-switchers did. Since participants in the two groups differed only in the frequency of daily code-switching, the different results may be related to the habit of switching between languages.

One of the bilingual language processing models that account for sentence context influence is the Bilingual Interactive Activation+ (BIA+; Dijkstra and Van Heuven, 2002). The BIA+ assumes that words from both of the bilingual’s languages are integrated in one lexicon in which activation is parallel and language non-selective. The fact that sentence context influenced reaction times to the French target words differently in low and high-constraint contexts is itself in support of the BIA+ assumptions regarding the influence of a sentence context on word recognition in bilinguals. The model assumes that sentence context affects word recognition through increased activation of items semantically related to the context. Because lexical activation is non-selective, all words that meet the semantic features evoked by the high-constraint context are activated regardless of their language membership. The activated words should then compete for selection. Competition is resolved by the top–down decision system that inhibits the task schema of the activated words in the non-intended language. In the case of the current study, words in Algerian Arabic and Standard Arabic should be inhibited in order for the bilingual to be able to name the words in the intended French language. This process should incur extra processing in code-switching the more strongly the anticipated word candidates are activated in the context language. Assuming that bilinguals build strong predictions in the highly constraining context, then competition between the activated words in the base languages and the words to be named in French would be strong and lexical selection through inhibition would incur extra processing load, leading to longer naming latencies. In this case, constraint effect is predicted to become smaller as naming latencies in the high-constraint context become larger. This is not supported by the present data which show reduced semantic constraint effects in the heavy code-switching group, but large effects in the light code-switching group due to facilitation effect in the high-constraint context. The finding that sentence context effects were different for the code switching groups is therefore somewhat problematic for this view.

The BIA+ model also contains a layer of two language nodes that function as language tags showing the membership of a word. The language nodes become activated late in the process and do not directly influence the lexical candidates. The model recognizes that the presence of a sentence context can pre-activate the language nodes, but because the language nodes cannot inhibit the non-target language words completely, sentence context cannot restrain language non-selective activation. The model does not clearly indicate the mechanism by which sentence effect takes place. With the assumption that the language nodes are activated late and cannot directly influence the lexical candidates, boosted semantic activation by itself is not enough to explain the different effect of sentence context in both groups. To account for the absence of cognate facilitation in the high but not in the low-constraint context, Schwartz and Kroll (2006) suggests that language nodes can be pre-activated by the highly constraining context. This may occur when the increasing constraining context attains an early stable activation in the lexicon which allows for an earlier activation of the language nodes. Even an additional assumption of the pre-activation of the language nodes through the increasing contextual constraint will not account for the different results in both code-switching groups. In that case, we should be looking for an explanation for why heavy code-switchers pre-activated the language nodes but not light code-switchers. In the rest of this paper, we will discuss how the findings in this study may be accounted for by a recent model of code-switching (Green and Wei, 2014).

The central idea of the control process model of code-switching is that language control varies depending on the different interactional contexts of the bilingual speaker and that the processes of language control can adapt to the demands imposed on them by these different interactions. The findings in the current study reveal that the effect of semantic constraint on the naming latencies to the French switches depended on the frequency of daily code-switching. Heavy code-switchers were slower to name the French NPs in both constraint conditions. In addition, the size of constraint effect was very small in bilinguals who frequently code-switch but was larger in bilinguals who do not code-switch frequently. This finding supports the general assumption of the control process model of code-switching that different language contexts induce different habits of language control. Shorter naming latencies in the high-constraint context may suggest that light code-switchers expected more a switch to the other language or that they integrated the switch more easily than heavy code-switchers. However, this interpretation sounds counterintuitive. Bilinguals who frequently code-switch should be more prepared to hear or integrate a switch. How can these results be interpreted by the control model?

The adaptive control hypothesis makes two important assumptions. The first states that experimental contexts can trigger the types of control processes in bilinguals. The prediction that follows is that the bilingual’s reactions to an experimental context can vary depending on how well the context fits the type of control processes for that bilingual. The second assumption concerns the individual differences. While bilingual speakers may experience more than one type of interactional contexts, their dominant type of language control is contingent on the typical exchanges that are recurrent within their speech of community. The model thus predicts that bilinguals who experience different interactional contexts may show adaptive responses that vary depending on how typical they are of each interactional context. The type of bilinguals tested in the current study are more representative of the dense code-switching in the Green and Wei model because they tend to adapt words morphologically as well as phonologically in informal contexts, although they may use French only during classroom hours, or use insertion in some other contexts. However, some of those participants are better representatives of dense code-switchers than others. The light code-switchers use both languages daily but do not frequently code-switch; their switches may be more regarded as insertions rather than integrated switches. The bilinguals who claimed they code-switch regularly may include more integrated switches than bilinguals who code-switch less frequently. In fact, some bilinguals asked during the training session whether they should read the target words in Algerian Arabic (meaning with Arabic phonology) or in French. Interestingly, even reminding the participants to read the targets in French did not eliminate few errors of the kind of phonological integration. In this case, those who frequently code-switch may be more familiarized with control processes that permit opportunistic planning, but those who do not code-switch frequently, and yet use both languages daily, may be more trained with interference suppression that taps on competitive relationship between the language schemas. On the other hand, the stimuli in the current study include code-switches that are in the form of insertion, baring no syntactic or phonological adaptation in the base language structure. When these stimuli are encountered, the language schemas are forced to consistently restrain from adapting the words. The stimuli may also require the participants to be in a coupled mode in which control passes from one schema to the other. In this case, the bilinguals who do not frequently code-switch may be more used to the type of stimuli presented in this study. The results revealed that light code-switchers showed larger semantic constraint effects that reflects facilitation of response in the high compared to the low context. This may suggests that the stimuli context triggered the control processes which the light code-switchers practiced more in their daily interactional contexts. By contrast, heavy code-switchers took longer time to name the switches and showed reduced semantic effects suggesting that facilitation in the high-constraint context did not occur. The stimuli should have forced them to engage control processes that are not typical of their interactional context. Heavy code-switchers had to control for the target words adaptation and engage a coupled control needed for insertion, whereas they are more used to an open control in which adaptation is allowed.

We turn now to consider the effect of base language. A main question in this study was to determine the effect of the languages involved in code-switching on the expectancy of a language switch. Comparison of effect sizes showed systematically a larger effect of sentence constraint in Algerian Arabic base language than in Standard Arabic base language. The results suggest greater expectancy of a switch when it is part of the typically conversational, recurrent code-switching. These results are even more important taking into account the relationship between Algerian Arabic and Standard Arabic. Out of 32 sentences, tested in this study, that occurred in the high constraining context 22 sentences were biased toward a target continuation that is shared between Algerian Arabic and Standard Arabic, assuming that the items are indeed predicted in the same language of the context. For instance, if a sentence context in Algerian Arabic constrained toward the Algerian Arabic *el baab* “the door,” a similar sentence context in Standard Arabic would bias toward the Standard Arabic *el baab* “the door.” It should be predicted that the participants’ reactions to the French translation NP *la porte* “the door” would be the same when listening to that sentence context in Algerian Arabic or in Standard Arabic. Given this information, one would not predict a difference between the two types of code-switching had the bilinguals anticipated the continuations in the base languages. In this respect, the differences in the naming latencies found between targets in the Algerian Arabic and Standard Arabic trials are to some extent meaningful. In particular, they suggest that it is possible for these bilinguals to anticipate a switch to L2 French when they listen to an Algerian Arabic context than when they listen to a Standard Arabic context. However, these results may be interpreted as ease of integration of a French switch when this later is part of the conversational code-switching.

The results concerning base language effect may be better understood by considering how the three languages are connected and stored in the bilingual memory. Both code-switching groups had moderately advanced level of proficiency in French and Standard Arabic. They acquired French at about 6/7 years of age and Standard Arabic at about 5. However, the groups differed in the frequency of code-switching. The findings suggest that in the light code-switchers, the French target item was already activated in the high-constraint context with the other translations in the base languages. When the bilinguals named the words in French they benefited from the early activation leading to a faster lexical retrieval. For the heavy code-switchers, activation of the French forms may not be as simultaneous as the other languages. Thus, naming the French targets would require extra time. The organization of lexical items may differ greatly depending on how the bilinguals represent the words semantically across the languages they speak (e.g., Basnight-Brown, 2014). There is a long line of literature showing that the ease of lexical retrieval depends on the degree of proficiency in L2 (e.g., Heredia, 1997; Heredia and Altarriba, 2001; Sunderman and Kroll, 2006; Kroll et al., 2010). When bilinguals become more proficient in L2, dominance may shift in some areas and words become more accessible in the language the bilinguals most often use (e.g., Cieślicka and Heredia, 2015). Results from the proficiency groups support this idea. Lexical retrieval in high proficient bilinguals was much faster than in low proficient bilinguals regardless of the context constraint, suggesting that activation is more automatic. However, proficiency by itself cannot account for differences among the code-switching groups in which bilinguals have about the same degree of proficiency, regardless of their code-switching habits. Language usage, and in particular the manner and the frequency of switching between languages, may have affected their lexical organization across these languages and hence lexical retrieval. Contrasting the present results with those from a study that tests heavy dense code-switchers on more typical stimuli may better determine the way these bilinguals store their words across the languages they speak.

To summarize, this study investigated code-switching processing in bilinguals who belong to a community where code-switching between Algerian Arabic and French is typical and dense. However, while these bilinguals differ in the amount and daily frequency of code-switching between Algerian Arabic and French they all claim that code-switching between Standard Arabic and French is not attested, not typical and find it rather odd to hear. During code-switching, bilingual speakers may anticipate a language switch. Expectancy of a language switch is more enhanced in a semantically rich context but also in a more typical context involving languages that are more frequently used in daily interactions. Anticipation of a language switch does not seem to depend solely on proficiency in the switch language. Results in the current study show that the ease of switching also depends on the habit and frequency of code-switching. These finding could be explained within the adaptive control hypothesis (Green and Abutalebi, 2013) and the control process model of code-switching (Green and Wei, 2014) which suggest that bilinguals’ daily habits of using their languages in different interactional contexts induce different habits of language control. It is worth noticing that although the switches in the stimuli are attested in speech, an ideal representative stimuli of dense code-switching would show integration of the code-switched items in Algerian Arabic base language. More representative stimuli of the heavy code-switchers would have enhanced the effect of base language and switching habits on the expectancy of a language switch. Unfortunately, such dense code-switching material cannot be tested using the current methodology because Algerian Arabic is not traditionally written. There are instances of written code-switching in social networks such as Facebook, however, it is written in French script and there is still controversy on how to spell certain Arabic sounds. A future improvement to this study would be to use auditory stimuli only with other techniques such as ERPs and eye tracking.

## Author Contributions

SK designed the study, collected and analyzed the data, and wrote the manuscript; EK directed the study, and was involved in the design and data analysis, and contributed to parts of the manuscript.

## Conflict of Interest Statement

The authors declare that the research was conducted in the absence of any commercial or financial relationships that could be construed as a potential conflict of interest.
